# Supplementary material for: Methanogenic patterns in the gut microbiome are associated with survival in a population of feral horses
Source: Nat Commun. 2024 Jul 22;15:6012. doi: 10.1038/s41467-024-49963-x (PMC11263349; doi:10.1038/s41467-024-49963-x)
Supplement: Supplementary file 1 — Supplementary Information [file 41467_2024_49963_MOESM1_ESM.pdf]

Supplementary Information for

Methanogenic patterns in the gut microbiome are associated with survival in a population of feral horses

Stothart, M.R.[1,2*], McLoughlin, P.D.[3], Medill[3], S.A., Wilson, A.J.[4], Greuel, R.J.[3], Poissant, J.[1*]

[1]Faculty of Veterinary Medicine, University of Calgary; Calgary, Canada.

[2]Department of Biology, University of Oxford, Oxford, UK

[3]Department of Biology, University of Saskatchewan; Saskatoon, Canada.

[4]Centre for Ecology and Conservation, University of Exeter; Penryn, UK.

*Corresponding author Email: masonstothart@biology.ox.ac.uk

*Corresponding author Email: jocelyn.poissant@ucalgary.ca

The PDF file includes:

Supplementary Notes 1 to 2

Supplementary Figures 1 to 9

Supplementary Tables 1 to 3

Other Supplementary Materials for this manuscript include the following:

Supplementary Data 1 to 4

Supplementary Note 1

Sable Island Horse Population Ecology. Horses were introduced to Sable Island in the mid-1700s[1]. In the intervening centuries Sable Island horses have been left free-living; although, additional intermittent introduction of horses from the mainland occurred as recently as 1940. Horses were similarly infrequently taken from Sable Island to be sold on the mainland until 1960, whereupon the Sable Island feral horse population was granted legal protections under the Sable Island Regulations of the Canada Shipping Act. Based on aerial surveys conducted over the decade after receiving protected status, the Sable Island horse population varied from 133–232 horses between 1961–1970[2]. In 2007, the population became the focus of a long-term individual-based ecological project. Ground-based surveys of the population during the 2013–2019 period represented in the present study estimate that population size has varied between 458–579 horses (n_{2013} = 559, n_{2014} = 552, n_{2015} = 458, n_{2016} = 510, n_{2017} = 511, n_{2018} = 558, n_{2019} = 579).

Horses occupy the entire vegetated length and width of the island. This population lacks predators, and so, is primarily bottom-up regulated by access to food and freshwater[2,3]. Nearly all mortality in this population occurs during the winter and early-spring, when horse energy reserves are low, and quality forage is unavailable. Marram grass is among the most abundant and widespread plant species on Sable Island and comprises a large portion of the horse diet; however, the semi-succulent plant sea sandwort is preferred where locally available at the eastern and western extremities of the island (pers. comm. K. Johnsen). Where permanent freshwater ponds are absent towards the eastern and western ends of Sable Island, horses are obliged to dig wells. The availability of freshwater ponds towards the longitudinal midpoint of the island influence horse movement, catalyze social interactions, and shape the social structure of the population, which segregates into 65–144 mixed-sex social bands or groups of bachelor males[4,5].

Mixed-sex social bands and bachelor male groups occupy spatially overlapping home-ranges. However, the Sable Island horse population is not freely inter-mix, but rather, segregates into two sub-populations in the summer at approximately the longitudinal midpoint, which are occasionally connected by migrants[6]. In the east, horse home-ranges are generally narrower and strongly influenced by the distribution of horse dug wells. In the west, horse home-ranges generally span the western third of the island and are shaped by the bi-directional movement of horses from sea sandwort lawns in the west, to freshwater ponds towards the island interior.

Sable Island horses have a polygynous mating structure wherein females segregate into mixed-sex social bands. Each harem of females is guarded by a dominant male (stallion), who monopolizes breeding opportunities by preventing mating incursions by bachelor males or competing band stallions. Males unable to secure a harem (a group of reproductively mature females) are solitary or form less stable social groups with other bachelor males. More rarely, sub-ordinate males are tolerated by band stallions if they participate in harem defense[7]. Between 50–80 mixed-sex social bands are observed on Sable Island annually, and across years, the average harem size is 2–3 females of reproductive age[4].

The population density of horses decreases from west to east on Sable Island[8], as does average horse body condition, intestinal parasitic nematode burden (estimated from fecal egg counts)[9], and the tolerance of horses to human presence[10]. Bacterial richness in the horse fecal microbiome similarly declines from west to east, however, horse access to low-fibre sea sandwort at the eastern and western tips of the island is also associated with a decrease in richness, as well as differences in fecal microbiome structure[11].

Supplementary Note 2

Sable Island National Park Reserve Description. Sable Island is a narrow oceanic sandbar, 160 km from the nearest landfall in eastern Nova Scotia, Canada[12]. The island is 1.5 km wide near the midpoint but stretches 42 km from east to west in near perfect perpendicularity to global meridians. Positions of longitude therefore provide an informative 1-dimensional proxy for spatial relationships on the island. Between 1801–1958, Sable Island was populated by a small community that operated a provincial life-saving station, which prevented shipwrecks, or rescued shipwreck survivors. Since 1958, Sable Island has lacked permanent human settlement, but has been intermittently peopled by Government of Canada staff and natural sciences researchers.

Three major environmental gradients dominate Sable Island:

(1) The Atlantic Ocean abuts contiguous sandy beaches on the north and south shores of the island along its entire length. Moving from shorelines towards the latitudinal midpoint of the island, barren beaches give way to American sea-rocket (*Cakile edentula*) dotted mounds that lay at the base of more densely marram grass (*Ammophila breviligulata*) and beach pea (*Lathyrus japonicus*) vegetated barrier dunes (10–30 m in height)[12]. Barrier

dunes to the north and south shelter late-succession heathland community valleys in the interior of the island[13].

(2) Sheltered heathland valleys and freshwater ponds are more common towards the longitudinal midpoint of the island, where the barrier dunes are tallest[14]. As the island tapers towards its longitudinal extremes, heathland valleys and barrier dunes give way to lower-lying marram and beach pea plateaus. Dense lawns of sea sandwort (*Honkenya peploides*) bookend the eastern and western tips of the island and are the only locations where this plant species is observed in abundance[14]; although, sea sandwort lawns were locally extirpated from the eastern side of the island by 2017, when a nearly 5 km segment of Sable Island became submerged by the Atlantic Ocean[12].

(3) Average dune height increases from west to east and vegetation density decreases along the same axis[15].

These environmental gradients combine to shape the population density, survival, fecundity, movement, adult sex ratio, social band structure, parasite load, behaviour, and gut microbiome of Sable Island feral horses[6,8–11,15,16].

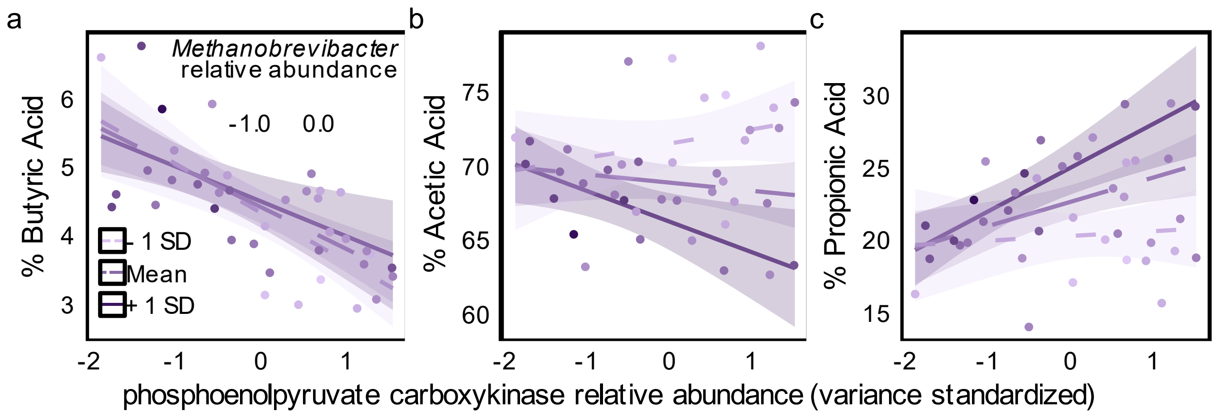

Supplementary Fig. 1. Percent abundance of primary short-chain fatty acids (SCFA) in relation to phosphoenolpyruvate carboxykinase gene hit relative abundance. Phosphoenolpyruvate carboxykinase gene relative abundance (centred and variance standardized) versus **a** % butyric acid, **b** % acetic acid, and **c** % propionic acid. General linear model (two-sided) predicted lines denote the effects that the mean (purple irregular dashed line), or a one standard deviation (SD) decrease (light purple dotted line) or increase (dark purple solid line) in *Methanobrevibacter* log-transformed relative abundance has on the relationship between phosphoenolpyruvate carboxykinase and % SCFA profiles, with 95% confidence interval shading. Phosphoenolpyruvate carboxykinase was negatively associated with % butyric acid ($\beta = -0.62\% \pm 0.12\%$, $t = -5.021$, $p < 0.001$), positively associated with % propionic acid ($\beta = 1.68\% \pm 0.58\%$, $t = 2.874$, $p = 0.007$), and not associated with % acetic acid ($p = 0.37$). Interactions between phosphoenolpyruvate carboxykinase and log-transformed *Methanobrevibacter* relative abundance affected % propionic ($\beta = 1.35\% \pm 0.57\%$, $t = 2.365$, $p = 0.02$) and % acetic acid ($\beta = -1.51\% \pm 0.59\%$, $t = -2.543$, $p = 0.02$), but not % butyric acid ($p = 0.39$). Effect estimates were obtained using two-sided general linear model tests of SCFA derived from 39 horse fecal samples. Source data are provided as a Source Data file.

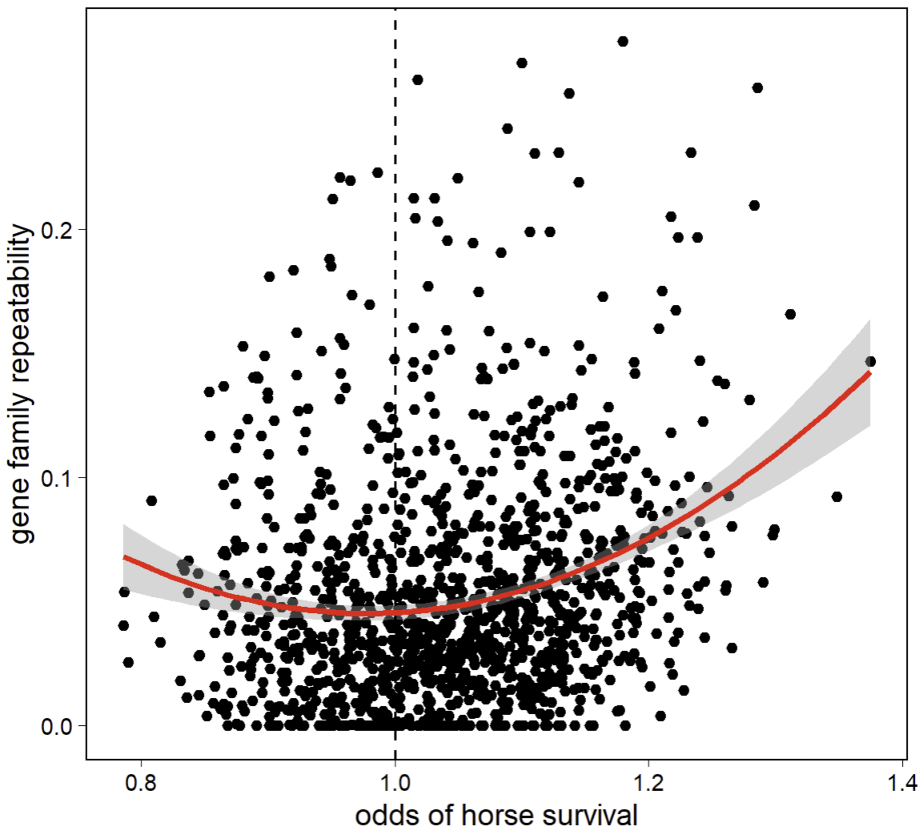

Supplementary Fig. 2. Patterns of repeatability in gene family abundance in the Sable Island horse fecal microbiome. Within-individual repeatability of centred log ratio (CLR) transformed gene hit abundance in relation to the estimated association between a one standard deviation increase in the CLR-transformed abundance of a given gene family and the odds of horse survival. Each gene family is represented by a single point. Solid red line denotes the best fit 2nd order polynomial from a two-sided general linear model (1416 gene families from 2394 samples spanning 794 individuals) with 95% confidence interval shading (linear term: $\beta = 0.30 \pm 0.04$, $t = 6.736$, $p < 0.001$; quadratic term: $\beta = 0.27 \pm 0.04$, $t = 6.109$, $p < 0.001$). Source data are provided as a Source Data file.

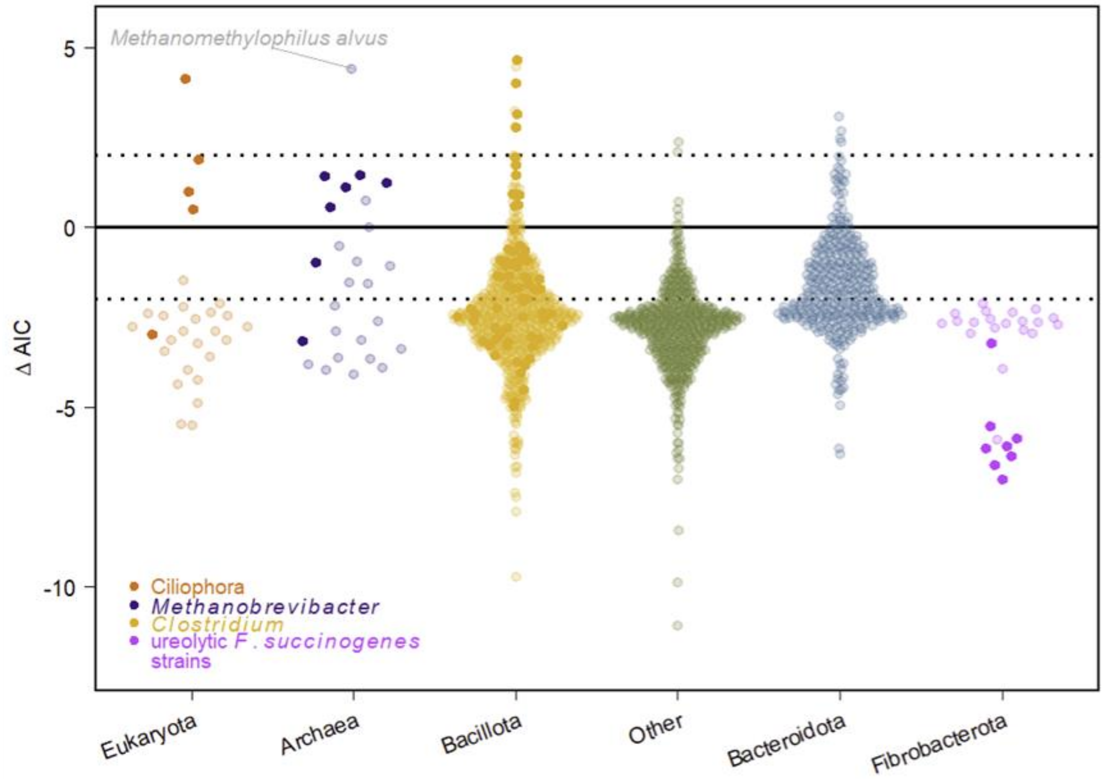

Supplementary Fig. 3. Akaike Information Criterion (AIC) comparisons of abrupt versus multi-year paradigms in fecal microbiome change in the years preceding horse death. △AICs ($AIC_{abrupt} - AIC_{multi\text{-}year}$) from two-sided generalized linear mixed effect model comparisons testing for abrupt versus multi-year changes in microbiota abundances preceding horse death (models containing fixed effects for either survival to the next year or years before death), with dotted lines denoting △AIC = 2 and −2, and negative values indicating stronger support for abrupt change model. A total of 1574 strain to phylum level microbiota are displayed. Models based fitted to 1127 samples spanning 418 individuals. Source data are provided as a Source Data file.

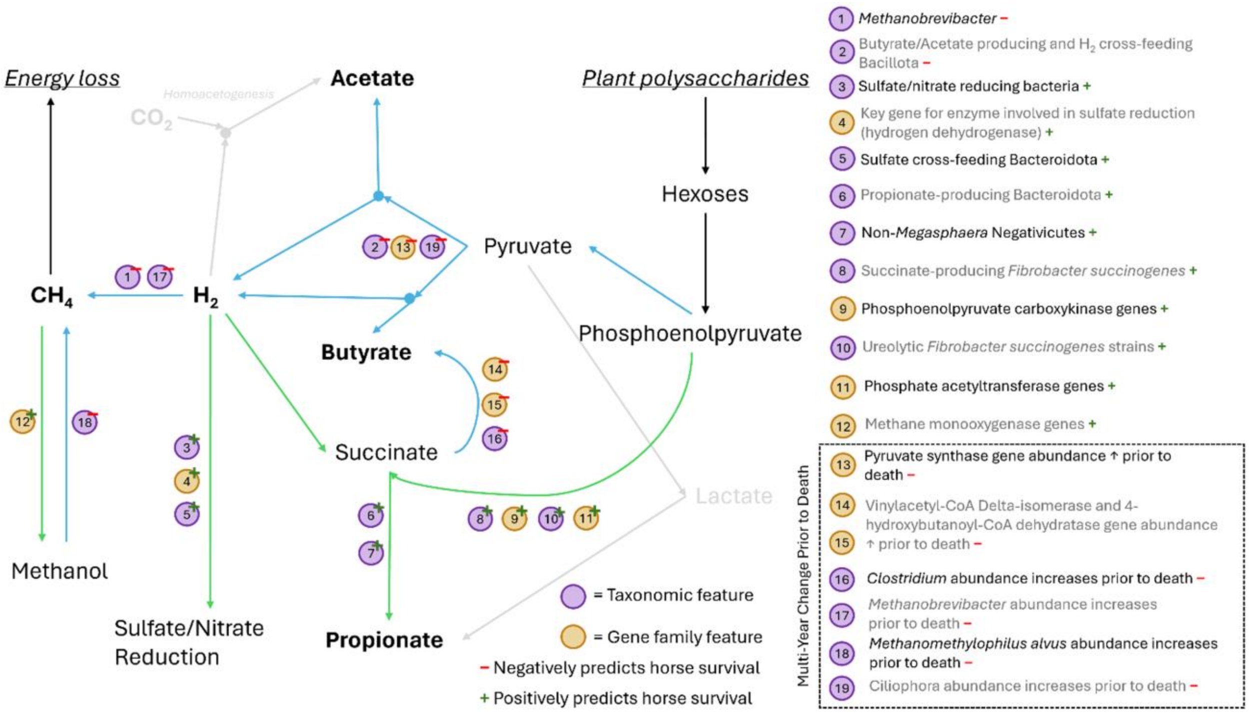

Supplementary Fig. 4. A schematic overview of our hypotheses of how metagenomic patterns associated with horse survival are connected to methane emissions from the horse hindgut. Purple circles denote taxonomic results, orange circles denote gene family results, blue arrows denote pathways generally expected increase methane production, green arrows denote pathways expected to decrease methane emissions, superscript + and – indicate features positively or negatively associated with horse survival, respectively. A dashed-line box separates features showing evidence for multi-year change preceding horse death. Grey pathways within the diagram highlight other non-methane hydrogen sinks hypothesized in the literature, but for which we did not find evidence.

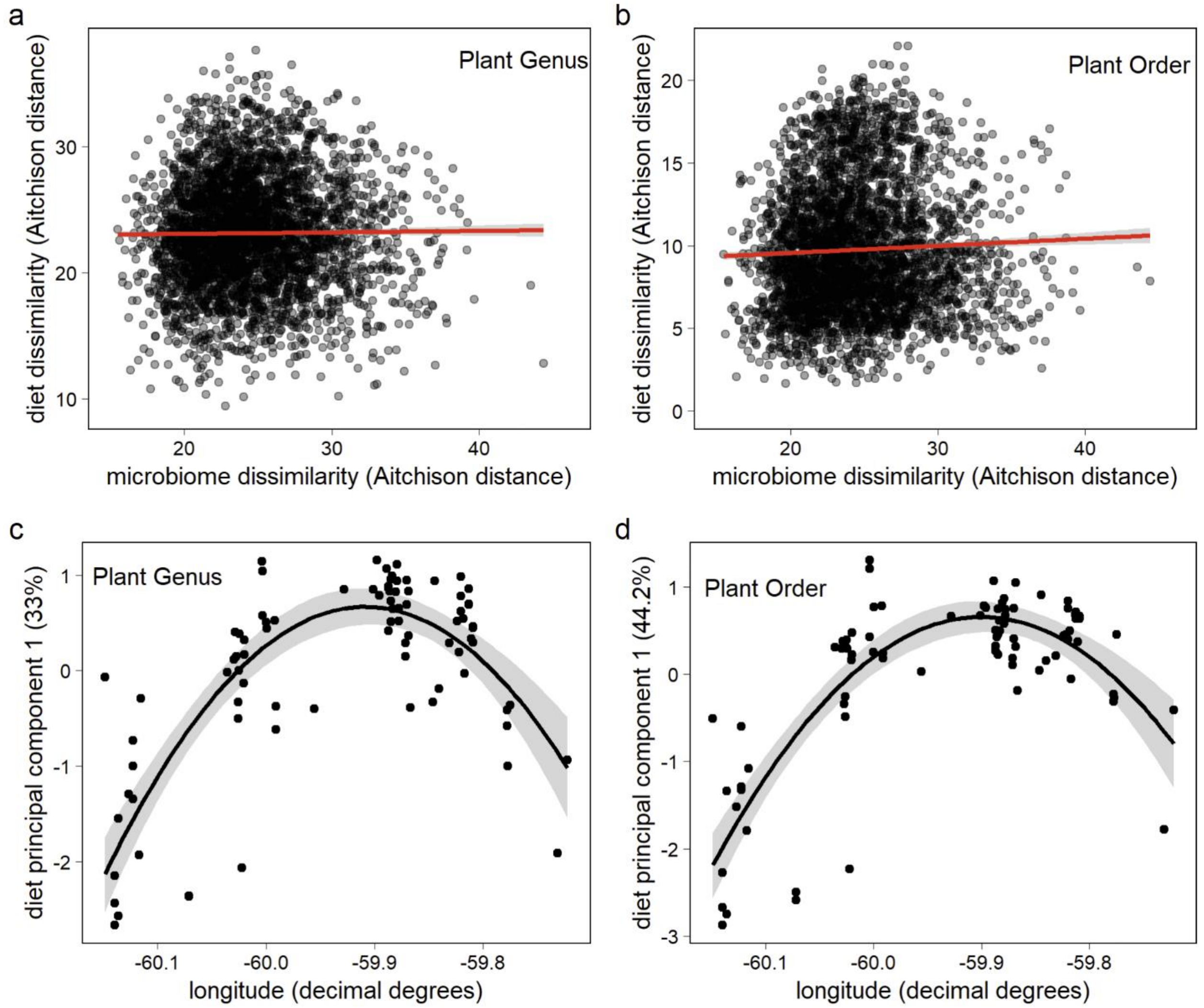

Supplementary Fig. 5. Patterns of variation in dietary metabarcoding profiles derived from Sable Island horse fecal samples. Aitchison distance dissimilarity in the horse fecal microbiome is not associated with Aitchison distance in diet profiles grouped to the levels of **a** genus (mantel test: $r_{pearson} = -0.009753$, p = 0.55, permutations = 9999) or **b** order (mantel test: $r_{pearson} = 0.04$, p = 0.28, permutations = 9999). General linear models reveal that longitude explains major variation in **c** genus-level ($\beta_{linear} = 4.72$, $t_{linear} = 7.553$, $p_{linear} < 0.01$; $\beta_{quadratic} = -5.445$, $t_{quadratic} = -8.709$, $p_{quadratic} < 0.01$) and **d** order-level (order: $\beta_{linear} = 5.30$, $t_{linear} = 8.856$, $p_{linear} < 0.01$, $\beta_{quadratic} = -5.16$, $t_{quadratic} = -8.618$, $p_{quadratic} < 0.01$) Aitchison dissimilarity in dietary metabarcoding profiles. Solid red lines are lines of best fit with 95% confidence interval shading. Solid black line denotes best fit quadratic relationships between longitude of sample collection and the first principal component from diet profiles. The data analyzed represents 85 paired metagenomic and dietary samples from 85 individuals. Source data are provided as a Source Data file.

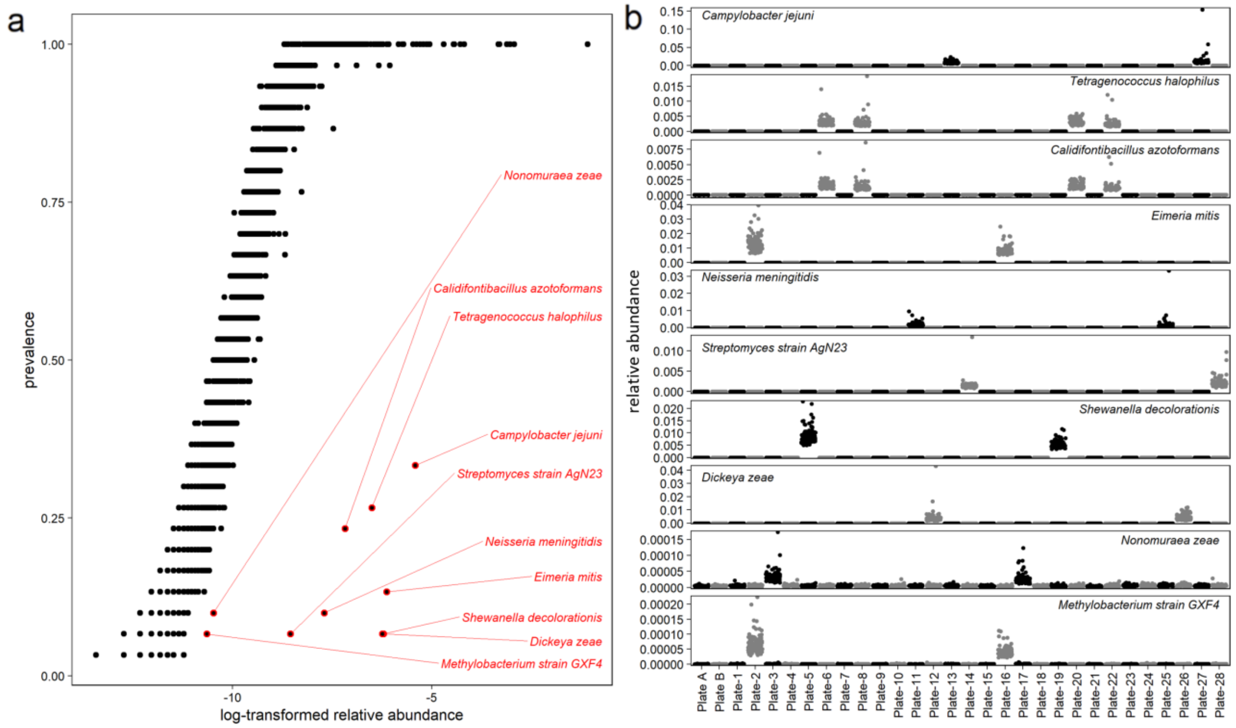

Supplementary Fig. 6. Identification of putative contaminants within the Sable Island horse microbiome dataset. a microbe average relative abundance versus prevalence across thirty ZymoBIOMICS Microbial Community Standard II (Log Distribution) positive controls with outliers labelled and highlighted in red. **b** relative abundance of outlier microbes within sequenced samples separated by library preparation plate. Data represents 2394 samples spanning 794 individuals. Source data are provided as a Source Data file.

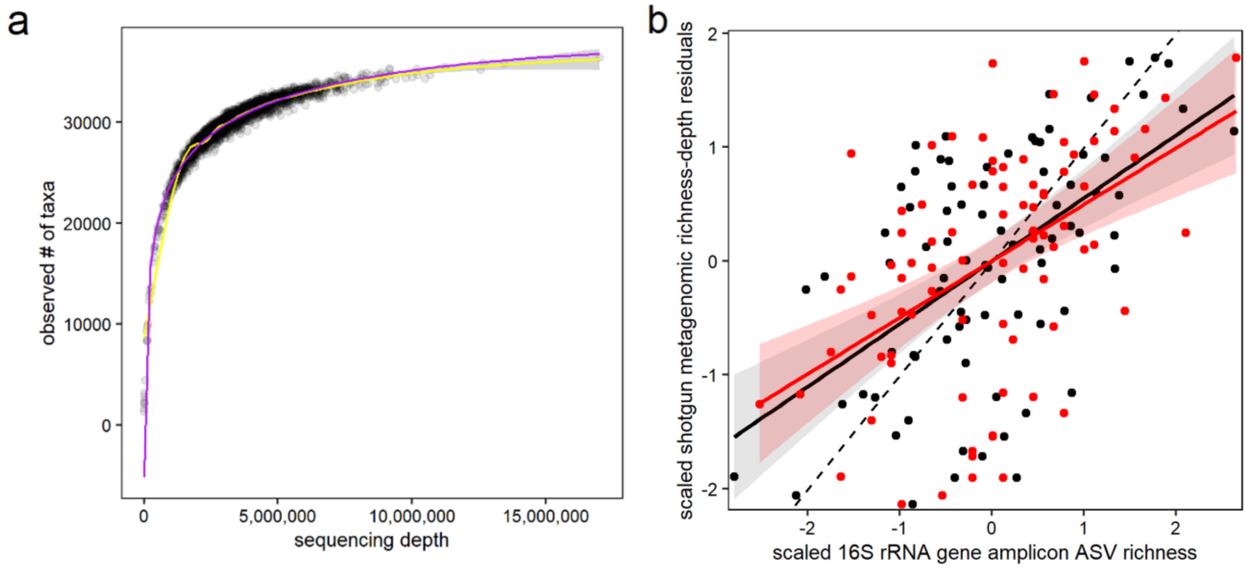

Supplementary Fig. 7. Derivation of richness estimates within the Sable Island horse fecal microbiome. a Shotgun metagenomic sequencing depth (after removal of unclassified reads) versus observed taxon richness with fitted line (purple: richness ~ log(depth) + depth) and locally weighted scatterplot smoothing fit (yellow) across 2394 samples spanning 794 individuals. **b** diversity-depth residuals from shotgun metagenomic sequencing data versus richness estimates obtained from paired 16S rRNA gene amplicon sequencing of the same DNA extracts (ASV-level: ●, genus-level: ●; 83 samples from separate individuals). Dotted diagonal denotes the 1:1-line, solid lines denote the line of best fit with 95% confidence interval shading. Source data are provided as a Source Data file.

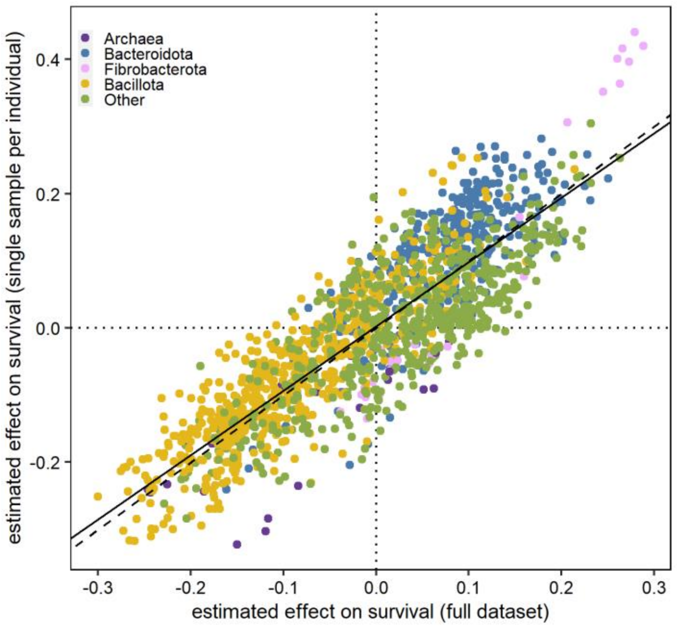

Supplementary Fig. 8: Associations of microbiota with Sable Island horse survival in the full versus single sample dataset. Estimates obtained from binomial generalized linear mixed models using the full 2394-sample dataset versus a model using only a single sample per individual (794 samples) in which survival was modelled as a function of microbiota centred log ratio transformed abundance. Solid line of best fit and a dashed 1:1 line. Microbiota (read pairs classified to the finest resolution possible, strain to phylum) are represented by points. Source data are provided as a Source Data file.

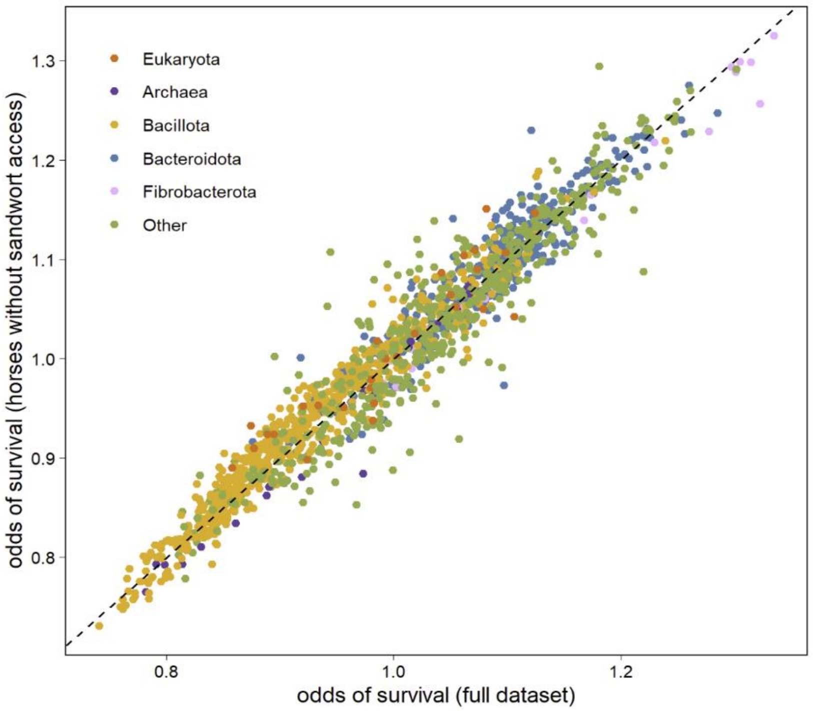

Supplementary Fig. 9. Associations of microbiota with the odds of horse survival from binomial generalized linear mixed models in the full dataset (2394 samples from 794 individuals) and among a subset of samples collected from east of −60.08º (2069 samples from 747 individuals). Dashed diagonal denotes the 1:1. Microbiota (read pairs classified to the finest resolution possible, strain to phylum) are represented by points coloured by major taxonomic grouping. Source data are provided as a Source Data file.

Supplementary Table 1. Output of a binomial generalized linear mixed model to estimate the effects of life history, spatial and microbiome community composition measures on Sable Island horse overwinter survival. Microbiome community composition is represented by the first six principal components (PCs) of an Aitchison distance ordination of microbiome community profiles. No adjustment for multiple comparisons in this two-sided model. This two-sided model was fitted to data from 2394 samples spanning 794 individuals.

term	model coefficient	std. error	z-value	p-value
taxonomic PC1	0.15	0.07	2.158	0.0309*
taxonomic PC2	0.11	0.07	1.66	0.0969
taxonomic PC3	0.04	0.06	0.661	0.5084
taxonomic PC4	−0.01	0.07	−0.178	0.8587
taxonomic PC5	−0.22	0.07	−3.317	0.0009*
taxonomic PC6	−0.16	0.06	−2.582	0.0098*
mean longitude (linear term)	−0.09	0.07	−1.422	0.1551
mean longitude (quadratic term)	−0.23	0.06	−3.674	0.0002*
age (linear term)	−0.31	0.07	−4.622	$3.80e^{-06}$*
age (quadratic term)	−0.34	0.06	−5.558	$2.74e^{-08}$*
sex	0.39	0.15	2.563	0.0103*
parental status (female)	−0.60	0.18	−3.277	0.0010*
parental status (male)	0.20	0.26	0.791	0.4290

* denotes statistical significance at $\alpha = 0.05$.

Supplementary Table 2. Output of a generalized linear mixed effects model to estimate the effects of life history, spatial and microbiome gene family composition measures on Sable Island horse survival. Microbiome gene family composition is represented by the first six principal components (PCs) of an Aitchison distance ordination based on read counts assigned to gene families. No adjustment for multiple comparisons. This two-sided model was fitted to data from 2394 samples spanning 794 individuals.

term	model coefficient	std. error	z-value	p-value
gene family PC1	0.16	0.07	2.45	0.0143*
gene family PC2	0.15	0.07	2.235	0.0254*
gene family PC3	−0.10	0.07	−1.395	0.1630
gene family PC4	−0.24	0.06	−3.768	0.0002*
gene family PC5	0.16	0.06	2.443	0.0145*
gene family PC6	−0.06	0.06	−0.915	0.3600
mean longitude (linear term)	−0.12	0.07	−1.814	0.0697
mean longitude (quadratic term)	−0.21	0.06	−3.625	0.0003*
age (linear term)	−0.29	0.07	−4.45	$8.58e^{-06}$*
age (quadratic term)	−0.36	0.06	−6.006	$1.90e^{-09}$*
sex	0.40	0.15	2.684	0.0073*
parental status (female)	−0.66	0.18	−3.59	0.0003*
parental status (male)	0.18	0.26	0.708	0.4789

* denotes statistical significance at α = 0.05.

Supplementary Table 3. AIC results from comparisons of the ability of variation in horse survival to be explained by a non-microbiome base model, a null model, taxonomic or gene family principal components (PC) models, and taxonomic or gene family 'distance-from-centroid' models. All binomial generalized linear mixed effects models included data from 2394 samples spanning 794 individuals.

Model	Fixed Effects	df	AIC	△AIC	*w*
Gene Family PC Model	base model + $PC1_{gene\ family}$ + $PC2_{gene\ family}$ + $PC4_{gene\ family}$ + $PC5_{gene\ family}$	13	1849.3	0	0.875
Community PC Model	base model + $PC1_{taxonomic}$ + $PC5_{taxonomic}$ + $PC6_{taxonomic}$	12	1853.2	3.894	0.125
Community Dispersion Model	base model + community dispersion	10	1867.5	18.177	< 0.001
Base Model	mean location + mean location^2 + age + age^2 + sex + sex:parental status	9	1871.9	22.579	< 0.001
Gene Family Dispersion Model	base model + gene family dispersion	10	1873.9	24.578	< 0.001
null model	intercept	2	1990.5	141.245	< 0.001

Supplementary References

1. Christie, B. *The Horses of Sable Island*. vol. 2 (Pottersfield Press, Lawrencetown, 1995).
2. Welsh, D. A. Population, behavioural, and grazing ecology of the horses of Sable Island, Nova Scotia. (Dalhousie University, Halifax, 1975).
3. Jenkins, E. *et al.* Not playing by the rules: Unusual patterns in the epidemiology of parasites in a natural population of feral horses (*Equus caballus*) on Sable Island, Canada. *Int J Parasitol Parasites Wildl* **11**, 183–190 (2020).
4. Manning, J. A. & McLoughlin, P. D. Environmental and demographic drivers of male mating success vary across sequential reproductive episodes in a polygynous breeder. *Ecol Evol* **9**, 5106–5117 (2017).
5. Manning, J. A. & McLoughlin, P. D. Climatic conditions cause spatially dynamic polygyny thresholds in a large mammal. *Journal of Animal Ecology* **86**, 296–304 (2017).
6. Contasti, A. L., Van Beest, F. M., Vander Wal, E. & McLoughlin, P. D. Identifying hidden sinks in growing populations from individual fates and movements: The feral horses of Sable Island. *Journal of Wildlife Management* **77**, 1545–1552 (2013).
7. Pinto, P., Mendonça, R. S. & Hirata, S. Examining the costs and benefits of male-male associations in a group-living equid. *Appl Anim Behav Sci* **253**, 1–12 (2022).
8. van Beest, F. M. *et al.* Increasing density leads to generalization in both coarse-grained habitat selection and fine-grained resource selection in a large mammal. *Journal of Animal Ecology* **83**, 147–156 (2014).
9. Gold, S. *et al.* Quantitative genetics of gastrointestinal strongyle burden and associated body condition in feral horses. *Int J Parasitol Parasites Wildl* **9**, 104–111 (2019).
10. Cabrera, D. *et al.* Island tameness and the repeatability of flight initiation distance in a large herbivore. *Can J Zool* **95**, 771–778 (2017).
11. Stothart, M. R. *et al.* Bacterial dispersal and drift drive microbiome diversity patterns within a population of feral hindgut fermenters. *Mol Ecol* **30**, 555–571 (2021).
12. Eamer, J. B. R. *et al.* Multi-decadal coastal evolution of a North Atlantic shelf-edge vegetated sand island — Sable Island, Canada. *Can J Earth Sci* **59**, 812–825 (2022).
13. Tissier, E. J., Mcloughlin, P. D., Sheard, J. W. & Johnstone, J. F. Distribution of vegetation along environmental gradients on Sable Island, Nova Scotia. *Écoscience* **20**, 361–372 (2013).
14. Rozen-Rechels, D. *et al.* Density-dependent, central-place foraging in a grazing herbivore: Competition and tradeoffs in time allocation near water. *Oikos* **124**, 1142–1150 (2015).
15. Contasti, A. L., Tissier, E. J., Johnstone, J. F. & McLoughlin, P. D. Explaining spatial heterogeneity in population dynamics and genetics from spatial variation in resources for a large herbivore. *PLoS One* **7**, 1–8 (2012).
16. Regan, C. E., Medill, S. A., Poissant, J. & McLoughlin, P. D. Causes and consequences of an unusually male-biased adult sex ratio in an unmanaged feral horse population. *Journal of Animal Ecology* **89**, 2909–2921 (2020).
