# Supplementary material for: Methanogenic patterns in the gut microbiome are associated with survival in a population of feral horses
Source: Nat Commun. 2024 Jul 22;15:6012. doi: 10.1038/s41467-024-49963-x (PMC11263349; doi:10.1038/s41467-024-49963-x)
Supplement: Supplementary file 3 — Reporting Summary [file 41467_2024_49963_MOESM3_ESM.pdf]

nature portfolio

Corresponding author(s): Mason Stothart

Last updated by author(s): Jun 4, 2024

Reporting Summary

Nature Portfolio wishes to improve the reproducibility of the work that we publish. This form provides structure for consistency and transparency in reporting. For further information on Nature Portfolio policies, see our Editorial Policies and the Editorial Policy Checklist.

Statistics

For all statistical analyses, confirm that the following items are present in the figure legend, table legend, main text, or Methods section.

n/a	Confirmed	
☐	☒	The exact sample size (*n*) for each experimental group/condition, given as a discrete number and unit of measurement
☐	☒	A statement on whether measurements were taken from distinct samples or whether the same sample was measured repeatedly
☐	☒	The statistical test(s) used AND whether they are one- or two-sided<br>*Only common tests should be described solely by name; describe more complex techniques in the Methods section.*
☐	☒	A description of all covariates tested
☐	☒	A description of any assumptions or corrections, such as tests of normality and adjustment for multiple comparisons
☐	☒	A full description of the statistical parameters including central tendency (e.g. means) or other basic estimates (e.g. regression coefficient) AND variation (e.g. standard deviation) or associated estimates of uncertainty (e.g. confidence intervals)
☐	☒	For null hypothesis testing, the test statistic (e.g. *F*, *t*, *r*) with confidence intervals, effect sizes, degrees of freedom and *P* value noted<br>*Give P values as exact values whenever suitable.*
☒	☐	For Bayesian analysis, information on the choice of priors and Markov chain Monte Carlo settings
☒	☐	For hierarchical and complex designs, identification of the appropriate level for tests and full reporting of outcomes
☐	☒	Estimates of effect sizes (e.g. Cohen's *d*, Pearson's *r*), indicating how they were calculated

Our web collection on statistics for biologists contains articles on many of the points above.

Software and code

Policy information about availability of computer code

Data collection	No software was used in the collection of this data
Data analysis	The bioinformatic tools Kaiju, Humann3 (DIAMOND), and Kneaddata (Bowtie2, Trimmomatic) were used to process shotgun metagenomic sequence reads. Dietary metabarcoding amplicons were processed using dada2 in R. All statistical analyses occurred in R using cited packages, and R code has been made available on figshare.

For manuscripts utilizing custom algorithms or software that are central to the research but not yet described in published literature, software must be made available to editors and reviewers. We strongly encourage code deposition in a community repository (e.g. GitHub). See the Nature Portfolio guidelines for submitting code & software for further information.

Data

Policy information about availability of data

All manuscripts must include a data availability statement. This statement should provide the following information, where applicable:

- Accession codes, unique identifiers, or web links for publicly available datasets
- A description of any restrictions on data availability
- For clinical datasets or third party data, please ensure that the statement adheres to our policy

Data Availability
The shotgun metagenomic sequence and dietary metabarcoding sequence data that support the findings of this study have been deposited in the NCBI SRA under

the BioProject accession codes PRJNA1102860 (https://dataview.ncbi.nlm.nih.gov/object/PRJNA1102860), PRJNA880353 (https://dataview.ncbi.nlm.nih.gov/object/PRJNA880353), and PRJNA1104620 (https://dataview.ncbi.nlm.nih.gov/object/PRJNA1104620). GC-FID SCFA data and sample metadata has been deposited on FigShare (10.6084/m9.figshare.25676670).

Code Availability
All code has been deposited on FigShare (10.6084/m9.figshare.25676670).

Research involving human participants, their data, or biological material

Policy information about studies with human participants or human data. See also policy information about sex, gender (identity/presentation), and sexual orientation and race, ethnicity and racism.

Reporting on sex and gender	This research did not contain data from human participants.
Reporting on race, ethnicity, or other socially relevant groupings	This research did not contain data from human participants.
Population characteristics	This research did not contain data from human participants.
Recruitment	This research did not contain data from human participants.
Ethics oversight	This research did not contain data from human participants.

Note that full information on the approval of the study protocol must also be provided in the manuscript.

Field-specific reporting

Please select the one below that is the best fit for your research. If you are not sure, read the appropriate sections before making your selection.

☐ Life sciences ☐ Behavioural & social sciences ☒ Ecological, evolutionary & environmental sciences

For a reference copy of the document with all sections, see nature.com/documents/nr-reporting-summary-flat.pdf

Ecological, evolutionary & environmental sciences study design

All studies must disclose on these points even when the disclosure is negative.

Study description	We opportunistically collected freshly defecated feces from feral horses on Sable Island National Park Reserve. In the research presented, we generated shotgun metagenomic sequence data from 2394 samples spanning 794 unique individuals.
Research sample	Sable Island feral horse feces (375 females spanning 1079 samples; 419 males spanning 1315 samples) collected from horses >= 1 year of age.
Sampling strategy	Sample size based on similar selection analyses conducted in other wildlife populations.
Data collection	Data were collected by numerous students, faculty, postdoctoral researchers, and field technicians over the course of 7 years.
Timing and spatial scale	Samples were collected from across the entirety of Sable Island National Park Reserve (Nova Scotia, Canada). Sample were collected across 7 years from mid-July to early-September, coinciding with feral horse population surveys as part of a longterm individual-based ecological study. The narrow sampling window helps to reduce seasonal confounds on the gut microbiome.
Data exclusions	Samples collected from foals were excluded from analysis, as the microbiome changes rapidly over the first few months of life, and we cannot accurately estimate foal age within this population at this time. 82 samples were removed from analysis because they did not achieve sufficient sequencing depth (based on thresholds defined in Stothart et al. 2021, Molecular Ecology Resources).
Reproducibility	No experiments were conducted in the course of this research. A small subset of samples (one sample per individual) were re-analyzed to test the robustness of biological signals. Results of this data subset were well-aligned with the full dataset (presented in the supplementary materials).
Randomization	Samples are opportunistically collected over the course of population surveys. In the event that a give individual had multiple samples collected within a single year, a sample was randomly chosen.
Blinding	Sample IDs are devoid of information about horse survival.

Did the study involve field work? ☒ Yes ☐ No

Field work, collection and transport

Field conditions	Fieldwork occurred on Sable Island National Park Research from mid-July to early September from 2013-2019. Daily mean temperature of 16C.
Location	Sable Island National Park Research 43.93325 N 59.89818 W
Access & import/export	Sample collection and laboratory analyses were performed under Parks Canada Agency Research and Collections Permit SINP-2013-2014, University of Saskatchewan Animal Care Protocol 20090032, and University of Calgary Animal Care Protocol AC18-0078.
Disturbance	Disturbance is minimal, as collections occur opportunistically. Researchers take care not to traverse dune faces (to prevent erosion) and use horse paths through the vegetation whenever possible.

Reporting for specific materials, systems and methods

We require information from authors about some types of materials, experimental systems and methods used in many studies. Here, indicate whether each material, system or method listed is relevant to your study. If you are not sure if a list item applies to your research, read the appropriate section before selecting a response.

Materials & experimental systems

n/a	Involved in the study	
☒	☐	Antibodies
☒	☐	Eukaryotic cell lines
☒	☐	Palaeontology and archaeology
☐	☒	Animals and other organisms
☒	☐	Clinical data
☒	☐	Dual use research of concern
☒	☐	Plants

Methods

n/a	Involved in the study	
☒	☐	ChIP-seq
☒	☐	Flow cytometry
☒	☐	MRI-based neuroimaging

Animals and other research organisms

Policy information about studies involving animals; ARRIVE guidelines recommended for reporting animal research, and Sex and Gender in Research

Laboratory animals	No laboratory animals were used in this research
Wild animals	Fecal samples were collected from feral horses (Equus caballus) opportunistically (no capture).
Reporting on sex	Findings in this paper pertain to both males and females. Horses can easily be sexed with cursory visual inspection (375 females spanning 1079 samples; 419 males spanning 1315 samples).
Field-collected samples	Collected samples were placed immediately on ice in the field, subset into 2-ml cryotubes within 6-8hrs, and frozen at -20C on Sable Island. Samples were then transferred to longterm storage at -80C. Samples were collected annually from 2013 to 2019, and collections covered the full spatial extent of Sable Island (Nova Scotia, Canada). Year-specific sampling windows were: 2013 (first = July 10th, last = September 8th), 2014 (first = July 21st, last = September 8th), 2015 (first = July 24th, last = September 1st), 2016 (first = July 22nd, last = August 29th), 2017 (first = July 17th, last = August 24th), 2018 (first = July 22nd, last = September 1st), 2019 (first = July 15th, last = August 25th).
Ethics oversight	Sample collection and laboratory analyses were performed under Parks Canada Agency Research and Collections Permit SINP-2013-2014, University of Saskatchewan Animal Care Protocol 20090032, and University of Calgary Animal Care Protocol AC18-0078.

Note that full information on the approval of the study protocol must also be provided in the manuscript.

Plants

Seed stocks	No seed stocks were used in this research
Novel plant genotypes	No novel plant genotypes were used in this research.
Authentication	No authentication was required, since no plants were used in this research.
