# Supplementary material for: Methanogenic patterns in the gut microbiome are associated with survival in a population of feral horses
Source: Nat Commun. 2024 Jul 22;15:6012. doi: 10.1038/s41467-024-49963-x (PMC11263349; doi:10.1038/s41467-024-49963-x)
Supplement: Supplementary file 4 — Description of Additional Supplementary Files [file 41467_2024_49963_MOESM4_ESM.pdf]

Description of Additional Supplementary Files

File Name: Supplementary Data 1

Description: Statistical outputs from two-sided binomial generalized linear mixed effects models used to estimate relationships between microbial taxon abundance (centred log-transformed) and Sable Island horse survival (2394 samples spanning 2394 individuals). Q-values reflect Benjamini and Hochberg false-discovery rate adjustment for multiple comparisons.

File Name: Supplementary Data 2

Description: Statistical outputs from two-sided binomial generalized linear mixed effects models used to estimate relationships between gene family hit abundance (centred logtransformed) and Sable Island horse survival (2394 samples spanning 2394 individuals). Q-values reflect Benjamini and Hochberg falsediscovery rate adjustment for multiple comparisons.

File Name: Supplementary Data 3

Description: Statistical outputs from AIC model competitions used to compare two-sided generalized linear mixed effects models for explaining variation in microbial taxon abundance (centred logtransformed) using either host survival (yes/no) versus years before death as explanatory variables. Benjamini and Hochberg false-discovery rate adjustments applied to features with △AIC values greater than zero. Modelled data represents 1127 samples spanning 418 individuals.

File Name: Supplementary Data 4

Description: Statistical outputs from AIC model competitions used to compare two-sided generalized linear mixed effects models for explaining variation in explaining variation in gene family hit abundance (centred logtransformed) using either host survival (yes/no) versus years before death as explanatory variables. Benjamini and Hochberg falsediscovery rate adjustments applied to features with △AIC values greater than zero. Modelled data represents 1127 samples spanning 418 individuals.
